# Annual Report of the Managers of the (N. Y.) State Lunatic Asylum, Made to the Legislature, January 31st, 1845

**Published:** 1845-05

**Authors:** 


1. *Annual Report of the Managers of the (N. Y.) State Lunatic Asylum, made to the Legislature, January 31st,* 1845. Albany, 1845.

2. *Twenty-fourth Annual Report of the Bloomingdale Asylum for the Insane, for the year* 1844. New York, 1845.

3. *The Annual Report of the Eastern Asylum in the city of Williamsburg, Virginia, for* 1844. Richmond, 1845.

4. *Annual Report of the Managers of the (Kentucky) Lunatic Asylum.*

1. The report of Dr. Brigham, like every thing else from his pen, is a well written document, and may be noted for brevity and accuracy of expression,—qualities, by the bye, which might be more studied by some of our writers of reports to the advantage of their readers, though the number of pages produced might in consequence be somewhat lessened.

At the beginning of the year, the number of patients in the Asylum was—

| | Men. | Women. | Total. |
|---|---|---|---|
| | 101 | 95 | 196 |
| Admitted since, | 132 | 143 | 275 |
| Total number during the year, | 233 | 238 | 471 |

Of this number there have been discharged—

| | Men. | Women. | Total. |
|---|---|---|---|
| Recovered, | 61 | 71 | 132 |
| Improved, | 26 | 21 | 47 |
| Unimproved, | 8 | 8 | 16 |
| Died, | 7 | 9 | 16 |
| Total discharged during the year, | 102 | 109 | 211 |
| Remaining November 30, 1844, | 131 | 129 | 260 |

In order to a correct understanding of these results, he explains what is meant by *recovered* and *improved* as follows:

"By recovery we generally mean a complete restoration of the mental powers. Most of those thus classed, when they left us, appeared not only to be sane, but to possess as sound and vigorous minds as before they were insane. But some few, though called recovered, and so considered by their friends, appeared somewhat eccentric or droll. They were not insane, and yet they could hardly be said to possess sound and vigorous minds. For the most part we find, on inquiry, they never had. Such individuals, after having had an attack of insanity, and been at an Asylum for the Insane and recovered, are ever after considered mentally deranged, though they are not, in fact, more so than they were previous to the attack.

"In some instances, though we are surprised at their rarity, the disease itself seems to have so impaired the physical system that the mind never fully regains its former power and activity, though the individuals become perfectly sane. On the contrary, the minds of some are actually improved by an attack of insanity. We have seen a considerable number of such cases, and we believe they will be more frequent when schools are generally established in Asylums, and books are plentifully and judiciously supplied to patients."

In 417 cases, in which the exciting causes of the disease are stated, the moral and physical are in the following proportions :—

*Moral.*—Religious anxiety, 77; loss of property, 28; excessive study, 25; death of kindred, 17; Millerism, disappointment in love, and other moral causes, 90. Total, 237.

*Physical.*—Ill health, 104; puerperal, 30; intemperance, 18; other physical causes, 28. Total, 180.

Among the novelties in the treatment of the insane, in which the prevailing spirit of the day is so fruitful, may be noticed a fair which was held at the Asylum the past year, and which is spoken of as follows:—

"Last year a fair was proposed, and the women engaged in it with such alacrity and spirit, that in a short time they made a great number of curious and beautiful articles; and in January, on the anniversary of the opening of the Asylum, the fair was held. From the sales at that time, and of articles made by the women and since sold, we realized a sufficient sum to purchase a considerable addition to our library; some musical instruments; a ticket of admission for all the inmates of the Asylum to the Museum in Utica for one year; and also to erect a handsome green-house. The green-house we find to be an admirable appendage to our establishment. Already we have in it four hundred flourishing plants, and it is the daily resort, in winter, of many of the patients.

"But the greatest good which resulted from the fair was, the pleasure which the devising and making of various articles afforded to many of the patients; and we hesitate not to say, that to several it was the means of restoration. Preparations for another are now in progress, with the like beneficial effects upon those engaged in it. We are confident that more good has resulted to our patients, from their mental and bodily labour connected with these fairs, than from all the various games and plays they have ever resorted to; and they have had recourse to many, such as battledoor, historical games, Dr. Busby's Mansion of Happiness, lottery games, Pickwick cards, &c. &c."

